# Using theory of change to co-create a programme theory for a telerehabilitation intervention for pain management in people with haemophilia

**DOI:** 10.1186/s13023-023-02988-9

**Published:** 2023-12-01

**Authors:** Paul McLaughlin, Michael Hurley, Pratima Chowdary, Kate Khair, Clive Smith, David Stephensen

**Affiliations:** 1 Katharine Dormandy Haemophilia Centre and Thrombosis Unit, Royal Free London NHS Foundation Trust, London, UK; 2 Department of Academic Haematology, University College, London, UK; 3 Kingston University, London, UK; 4 Haemnet, London, UK; 5 The Haemophilia Society, London, UK; 6 School of Medicine, Health and Social Care, Canterbury Christchurch University, Canterbury, Kent UK

**Keywords:** Rehabilitation, Telerehabilitaion, Haemophilia, Chronic pain, Programme theory, Co-creation, Theory of change

## Abstract

**Background:**

Improved approaches for chronic pain management are a clinical and research priority for people with haemophilia (PWH). Involving people with lived experience in the design of a complex rehabilitation intervention strengthens the credibility and plausibility of the intervention, particularly in relation to rare disorders. Here we describe using a ‘Theory of Change’ (ToC) dialogue-based stakeholder process to create a programme theory for a telerehabilitation intervention.

**Methods:**

An online workshop was convened and stakeholders received a briefing document in advance. Five stakeholders took part (3 PWH and 2 physiotherapists). At the workshop the group first agreed the overall aim of the intervention. Discussions then identified the resources, activities, barriers and enablers needed to achieve this outcome. All discussions were recorded and annotated by the workshop moderator. Behaviour change techniques were mapped for inclusion in the theory.

**Results:**

A programme theory and narrative report were produced. All stakeholders reviewed these for clarity and to ensure a true reflection of the workshop discussions. Agreement was based on how meaningful, well-defined, do-able, plausible, credible, and testable each component was. Stakeholders highlighted the importance of issues unique to PWH. Key components included the need for physiotherapists to be knowledgeable of the condition, a range of exercises that were inclusive of all abilities, and the need for people to feel safe and supported whilst taking part.

**Conclusions:**

Co-developed theory based approaches to intervention design offer an inclusive and transparent way to develop novel and meaningful interventions for people with complex health conditions. The ToC is wholly transparent in its design and content. Together with the identified behaviour change techniques, the theory informs the protocol for a feasibility study evaluating a telerehabilitation intervention. Importantly, it allows the opportunity to revise, adapt and improve the programme theory for further implementation and evaluation.

## Background

Haemophilia is an umbrella term for the most common of the rare lifelong bleeding disorders, which in its untreated state can result in spontaneous musculoskeletal bleeding [1]. Recurrent episodes of bleeding into joints over a lifetime leads to the development of a painful haemophilic arthritis, usually affecting the elbows, knees and ankles [2]. Chronic pain as a result of this haemophilic arthropathy presents a significant physical, social and personal issue for people with haemophilia (PWH) [3], and one that many feel is poorly managed by haemophilia clinical teams [4]. Recent guidelines for the management of chronic pain in PWH adopt a predominantly biomedical view of escalating pain medications [5], and there remains a significant limitation in high quality evidence of effectiveness of many physiotherapy interventions for pain in PWH [6]. Despite good evidence of effect of exercise-based rehabilitation as a primary intervention for pain in other arthritides such as osteoarthritis and rheumatoid arthritis [7, 8], little is understood about the potential effectiveness of such an approach in PWH. Such limitation is further compounded by the experiences of historical medical management of haemophilia, which often explicitly linked exercise as a risky behaviour that provoked bleeding [9]. This current landscape provides a clear rationale for the need to engage PWH in the development and design of rehabilitation interventions that aim to help manage the burden of living with chronic pain.

The recently updated UK Medical Research Councils (MRC) framework identifies theory development and stakeholder involvement as core elements of complex intervention development [10]. Awareness of the contexts and constraints in which an intervention may be operating is also an important consideration in complex intervention development [11]. Meaningful engagement of stakeholders in theory development maximises the probability of developing an intervention that delivers meaningful and positive impacts on health and can help identify critical aspects of an intervention and how they may be related, further strengthening real-world applicability [10, 12].

Programme theory is the construction of a plausible and sensible model of how an intervention is supposed to function, is practical and specific to each intervention, and justifies the intervention in terms of its expected casual effects [13, 14]. By starting with a programme theory, it is made clear just how many and varied the processes are that may lead to an intervention’s success or failure [15]. Without it, it is impossible to know if the aspects of implementation quality and quantity have been measured correctly [16]. Programme theory can be expressed as process models, logic models or frameworks, and their purpose is to summarise key programme elements that include the programme assumptions, programme activities as well as the inputs, outputs and outcomes [17].

One approach to theory development is that of Theory of Change (ToC), defined as a theory of how and why an initiative works. It should involve stakeholders, and combines logical thinking (sequencing an initiative from inputs to outcomes) alongside deep critical reflection of values, worldviews, assumptions and philosophies as to why and how a change may happen because of the intervention [18]. More often associated with large international development projects, in recent years healthcare researchers have begun to use this process approach in the development and evaluation of interventions [19, 20]. The ToC approach works well when developing a complex intervention and privileges the views, beliefs and experiences of the actors involved in the intervention (the designers, those that receive it and those that will deliver it). As a pragmatic framework, ToC complements the intervention development phase of the MRC framework, and importantly, can accommodate other theories to explain causal links and improve development of research projects [20]. The minimum elements that should be included in a ToC are:

- context defined and acknowledgement of existing change processes and the actors able to influence change
- The long-term change that the intervention seeks to support and for whose benefit
- The processes and sequence of change anticipated to lead to the desired outcomes, and assumptions about how these changes might happen
- A diagram and narrative summary that captures the outcomes of the discussion, providing further explanatory detail about the ToC as well as highlighting any elements that are not included in the model itself [18]

Other mid-range social or psychological theories such as behaviour change theory (e.g. the behaviour change wheel and COM-B model) [21] can be integrated into a ToC framework. In doing so it can strengthen the explanations for observed causal relationships, increasing the ability to explain ‘why’ and ‘how’ an intervention has its effects. In developing complex rehabilitation interventions that aim to elicit behaviour change, there is a need to use a method that incorporates an understanding of the behaviour to be changed and uses a system that characterises the interventions and their components [21]. Theories of behaviour change can help identify barriers and facilitators to change as well as mechanisms of action.

To develop complex interventions which are more likely to be effective, sustainable, and scalable, it is important to understand how and why the intervention has a particular effect, and which parts of a complex intervention have the greatest impact on outcomes.

The aim of this study is to describe the development of a programme theory using a theory of change approach. This programme theory mapped alongside behaviour change techniques, will then provide the basis for a theory-driven evaluation of the feasibility and acceptability of a future telerehabilitation intervention for PWH living with chronic pain.

## Methods

The stakeholder group was formed by people who approached the authors expressing an interest to take part (after hearing about the overall project) and others who had participated in a previous qualitative study. Participants were sent a detailed description of the workshop aims and objectives, the time commitment required from them, and a brief description of how the workshop would be run. They also received a copy of a briefing document which included a synthesis of current literature on the topic and an analysis of need for the development of this programme theory [22]. Participants were asked to read this document before the meeting, to formulate their own views and opinions of the document and consider how it could help inform the ToC process.

Stakeholder workshops for a ToC process are usually in person which allow for a high level of interaction to conceptualise, organise, and agree ideas that ToC is associated with. However, due to the ongoing COVID-19 restrictions regarding face-to-face meetings at the time, the workshop was convened using an online meeting platform (Zoom®). The workshop was held on the 4^th^ November 2020, with a lead facilitator (PML) and a second moderator in attendance (DS). To try and create as open and interactive a session as possible, an online platform called Padlet® was used (www.padlet.com). Padlet is a real time, collaborative platform that functions like a digital white board. Users can create walls upon which notes can be added, and it also supports other file types such as pdfs, word documents and pictures. These notes can then be moved, connected, removed, and edited as group discussions progress. The meeting host (lead author) facilitated the screen-share of a blank Padlet screen with all of the participants. The lead author was responsible for typing everything that was discussed onto the Padlet wall.

As a truly stakeholder-led, iterative approach ToC does not require the use of an a-priori framework or topic guide. Only the overall purpose of the approach was stipulated upfront, that is, to create a theory that would underpin the development of study protocol for a telerehabilitation intervention. The stakeholders used their own lived experiences and expertise to interpret the briefing document to then identify and agree what the overall aim/ long term change for a rehabilitation intervention for pain management would be, i.e., the end point. From this point the group works ‘backwards’ identifying the medium and short terms outcomes, the pathways needed to achieve the outcomes, as well as the people, places and contextual issues involved at each stage. The overall discussion points were focussed on the exercise-based intervention delivery, content, logistics, barriers and enablers. Participants were encouraged at all times to be mindful of the transparency of proposed causal attributions as well as being realistic in how their suggestions happen in a real-world setting. Notes were made as participants discussed them, making sure that queries were addressed about accuracy and meaning if it was unclear. Participants were encouraged to keep looking and thinking at processes to enable the change they wanted to see in the intervention, the ‘how and why’ rather than just the ‘what.’ Two Padlet walls with notes were produced from the discussions in the group. The workshop was recorded on Zoom® with permission of all attendees. This allowed for the Padlet creation process in the workshop to be further be reviewed, analysed and edited afterwards.

In the week following the workshop the lead author produced a preliminary programme theory model and narrative summary of the ToC process. This was then sent to the stakeholder group for review and comments/additions/edits. Within two weeks, all of the group had responded, and the model was reviewed and edited accordingly. When all of the group had agreed the model and report represented the workshop and the process overall, was it signed off as complete. The PWH stakeholders were reimbursed for their time.

**Fig. 1:**
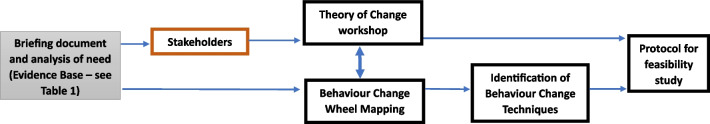
Schematic overview of the process of Theory of change creation and mapping of behaviour change techniques

A behavioural diagnosis was completed using the COM-B behavioural diagnosis form [23]. The output of the ToC process was mapped against the COM-B model and using the Behaviour Change Wheel, relevant intervention functions were identified [21]. Specific behaviour change techniques (BCT’s) were then chosen from the BCT Taxonomy [24] and linked to these intervention functions, targeting potential mechanisms of change to include and evaluate within the proposed feasibility study (Fig. 1).

## Results

Three male persons with severe haemophilia A (mean age 36.3 years, range 27–53), and two female specialist haemophilia physiotherapists (with over 25 years combined experience in haemophilia) volunteered to participate in the TOC workshop. The workshop lasted 3 h including a 20 min break.

**Fig. 2:**
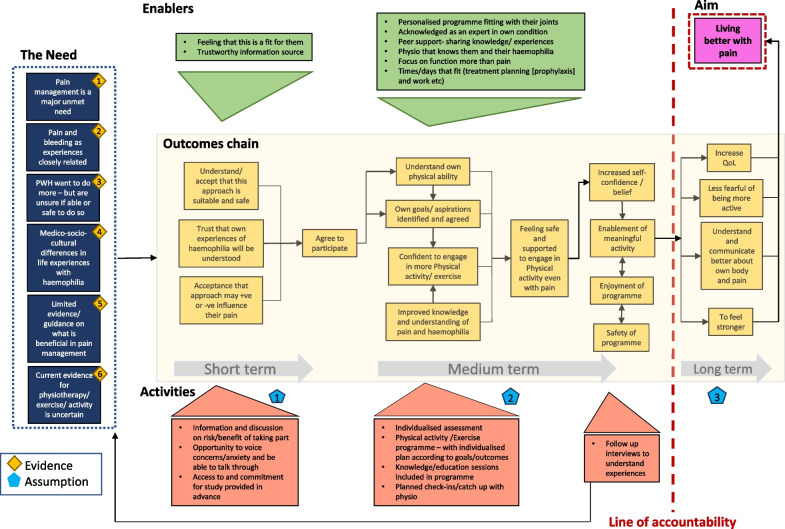
Theory of change model

The final theory of change model is presented below in Fig. 2.

The overall aim of the intervention described by the stakeholders was ‘Living better with pain’ and is presented on the top tight hand side of the model. The ‘need’ for this approach based on the current available evidence, is summarised, and presented on the left of the diagram. The ‘outcomes chain’ relates to the period of the planned feasibility study and presents the assumed causal chain of events for a participant to successfully take part in the intervention. The successful progression through the outcomes chain is dependent on the influence of the identified ‘enablers’ and the inclusion of ‘activities’ within the intervention itself. The ‘line of accountability’ highlights the longer-term outcomes that are considered beyond the scope and measurement of the planned study. The assumptions and evidence are presented in Table 1.

**Table 1:** Evidence base and assumptions included in the Theory of Change

Assumptions	1. - PWH who have pain attend regular clinical reviews in their haemophilia centre- so will be identifiable as possible participants from there - Physiotherapists will be engaged in the study and willing to undergo training to deliver the intervention - Information about the study will be delivered by the specialist physiotherapist known to the PWH 2. - The technology will be available for both PWH and Physiotherapist to participate virtually in real-time in this study - Training will be provided for PWH on using virtual/ remote digital platforms - Intervention activities will be carried out within normal working hour week - Outcome measures will be selected for the study protocol informed by qualitative studies and this theory of change workshop 3. - The map created here will likely need to change/be adapted following completion of the study—this will be addressed in a follow up Theory of Change meeting
Evidence	1. Literature review (Overview of key findings from narrative review of literature relating to haemophilia, pain, exercise and rehabilitation) 2. Systematic Reviews [6, 25] 3. James Lind Alliance Report [26] 4. Findings of previous qualitative studies [9, 27] 5. World Federation Haemophilia Guidelines [5]

## Theory of change narrative summary

### Context and need

Chronic pain associated with haemophilic arthropathy is a pressing clinical and personal issue for many PWH [26], with figures indicating a prevalence of between 40 and 46% of PWH experiencing chronic pain [28, 29]. Pain in in haemophilia is complex due to its close association with bleeding, its lifelong presence, and the difficulties with acute on chronic pain episodes experienced by those with living with haemophilic arthropathy. Fear of bleeding as an initial response to pain has meant that many living with pain feel unwilling to be more physically active in case of further consequences. However, PWH acknowledge they would like to feel more confident and better supported to be more active and functionally well regardless of their pain.

Whilst guidelines relating to pain management in haemophilia relate mostly to pharmacological/drug management, qualitative findings suggest PWH are unwilling to take further pain medications and would rather seek out other non-pharmacological options. Individual life experiences of haemophilia are varied and depend on age and availability of treatment, country of birth and access to knowledgeable haemophilia healthcare professionals. Such life experiences of healthcare, as well as living and managing with a rare disease, have meant many PWH are experts in their own disease on a day-to-day basis.

### Activities/strategies

A major determinant for successful implementation of the feasibility study will be to manage an individual’s initial fear and anxiety around such an approach and to embed an engaging ‘sales pitch’ as part of the recruitment. A recurring theme highlighted by stakeholders was the importance of acknowledging the variance of joints affected by haemophilic arthropathy across possible participants and to provide reassurance on its appropriateness for someone like them. Individualised assessment and development of a personalised programme would be a key component of participation and be completed by a physiotherapist that knows them and their haemophilia.

A focus on increasing physical activity levels and meaningful physical function, of which ‘exercise’ can be part, was felt to be more acceptable to PWH, whilst still being able to capture and engage those with a wide spectrum of physical ability, pain, and joint disease. Sessions delivered by a trusted source will have a core element of cardiovascular activity and functional strengthening. Approaches such as High Intensity Interval Training (HIIT) were discussed, as the short burst nature of its application can work well for those easily fatigued and with joint pain issues. However, the group agreed that a low impact, moderate intensity activity was more appropriate to accommodate for all fitness and joint health levels. The programme should accommodate progression of activity over the duration of the study, with regular check-ins and reviews helping engagement.

Knowledge and discussion (rather than education) sessions will be interspersed throughout the intervention. These will focus on understanding joint health in relation to bleeding, arthritis, and pain, as well as information about the benefits and safety of physical activity even with haemophilia and arthritic pain. This was proposed as a way of enabling PWH to be able to understand and reason better about their own pain experiences, regaining control over their pain limited activity and being more confident in their decisions to be active with pain.

### Enablers

Successful impact on the outcomes chain from the activities will be influenced by identification of enablers and barriers to implementation. The group identified that the focus of the intervention should be on better day-to-day function rather than just measuring pain, helping people focus on things they feel they can change. It was highlighted as important that PWH feel that they ‘belong’ in an approach that uses physical activity and exercise as an intervention. This is embedded in the lived history of their experiences of activity, bleeding, and pain, as well as what they have been advised growing up about exercise (i.e., possibly dangerous).

Conversations and discussions about participating in this type of study need to be held with people they trust and know, such as a known and trusted haemophilia specialist physiotherapist. People with haemophilia need to be acknowledged as experts in the day-to-day management of their condition and for this to play an active role in conversations around goals and outcomes of participating in a study like this. An individual assessment and personalised plan to use in the study will facilitate engagement and allow positive discussions around why this kind of intervention will be ok for them to do.

Worries about the possibility of bleeding due to being more active can be managed if the time commitment, days and times of sessions are known in advance and prophylaxis treatment schedules instigated. Having confidence in a high circulating factor level (when pain is present) allows people to be less concerned about ‘bleed pain’ and focus more on arthritic pain, which is viewed as less threatening. Planning for participation is important so that it can also be accommodated into the working day, as many PWH are of working age.

Anxiety over negative body image and worries of feeling intimidated about doing exercise activity will be minimised by doing it in a virtual 1:1 session. However, having an opportunity to share thoughts and experiences with others may be a positive element of the intervention and bring the benefit of social connection by discussing shared-experiences and strategies that others have used and can use. Feeling part of a group in this way may also increase motivation to continue with the programme by fostering a sense of shared identity and experience.

### Desired results (outcomes chain)

The outcomes chain identifies outcomes across the short and medium time frame, with interconnectedness between outcomes highlighted by directional connecting arrows. The short-term outcomes relate to study recruitment, and demonstrate the consequence of the personalised and highly informative process in getting people to consider participating in the study. Improved understanding of why and how a programme like this may be beneficial for them will facilitate a process of well-informed internal reasoning resulting in agreement to participate.

The medium-term outcomes relate to active participation in, and delivery of, a physiotherapy led programme of exercise. It highlights how feeling safe and confident to engage in physical activity with pain present is influenced by individualised physical capabilities of fitness and strength, as well as enhanced knowledge and understanding of pain and arthropathy that relates to them and their life experience. An improvement in self-belief, improving physical ability, experiencing the safety of the intervention and enjoyment of the programme leads to further participation in activities that matter to them outside of the study activity.

### The line of accountability

At the “line of accountability” on the ToC map it cannot be implied that the intervention described is driving change in long term outcomes anymore. The proposed feasibility study will aim to see if PWH will participate in this intervention, if it is acceptable and safe for then to do so, and if activities in the study have any short- to medium-term effect on self-identified outcomes relating to physical function and day to day pain. It is unclear if longer term health benefits can be achieved with this study approach alone.

### Mapping COM-B to the theory of change map

The behavioural analysis focussed on how to engage PWH with chronic pain to take part in an exercise intervention delivered virtually in real-time by their specialist physiotherapist. A behavioural diagnosis was completed using the COM-B behaviour diagnosis form. This clarified and quantified the specific details for mapping to the COM-B and helped identify the intervention functions of interest. These intervention functions then identified 21 BCT’s to be included in the design of the feasibility study protocol. These BCT’s are detailed in Table 2 and listed according to their corresponding number in the BCT Taxonomy [24].

**Table 2:** List of behaviour change techniques included in design of feasibility study

BCT label and taxonomy number	Intervention component
1.1—Goal setting (behaviour)	For participants to participate in a virtual online exercise session twice a week. Sub goals based on self-identified activities of choice
1.2—Problem solving	Identify potential barriers to taking part in the study and generate individualised solutions to help overcome barrier—can be iterative process as intervention proceeds. Group knowledge and discussion sessions used to work through barriers or negative emotions/thoughts in respect of activity
1.3—Goal setting (outcome)	To be more willing to be more active (even with pain) at the end of the study
1.4—Action planning	Intensity of intervention determined from initial visit with physiotherapist. Sessions planned in accordance with established prophylaxis regime (safety planning). Discuss with each participant what their trough levels would be the day after prophylaxis (enable reasoned process if pain worse and fear of bleed). Encouragement to wear splints, supports etc. if they feel they need to
1.5—Review behaviour goal(s)	Weekly review of attendance and participation in intervention—amend/revisit initial goals setting if newly identified issues
2.2—Feedback on behaviour	Feedback and recap by physiotherapist at end of each week’s activity
2.3—Self monitoring of behaviour	Weekly reflective diary on own activity and pain—to include RPE scale with activity each week
2.7—Feedback on outcomes of behaviour	Feedback at study end of before and after results of outcome measures
3.2—Social support (practical)	Participant partner/family/neighbour will be used if necessary to help in setting up the webcam if necessary—as well as being someone to call upon if any issues such as risk of falls etc
4.1—Instruction on how to perform behaviour	Participants will have practice session for exercises on their list—as well as set up instructions for webcam visuals, plus using diary
5.1—Information about health consequences	Providing information on joint damage and pain in haemophilia and benefits of physical activity/exercise
5.4—Monitoring of emotional consequences	Encouraged to discuss worries/fears whilst taking part in study (focus on pain and activity) and weekly diary
6.1—Demonstration of the behaviour	Each exercise set and start point will be agreed upon at initial visit with explanation and practice demonstration within boundaries of individual ability. Physiotherapist will demonstrate each exercise within the session before each set
7.1—Prompts and cues	Laminated RPE scale next to webcam so can look and answer physiotherapist when asked about this in activity session
8.7—Graded tasks	Exercise activity has graded allowances built-in (more reps, harder/easier effort level)—and will be increased weekly depending on performance
9.1—Credible Source	Intervention will be delivered by expert haemophilia physiotherapist, known to the participant
9.2—Pros and cons	Discussions in initial visit to encourage individual to identify pro’s and con’s to taking part in this study—which are noted in the individual case report form and discussed with physiotherapist
12.1—Restructuring the physical environment	Encouraged to create a place of quiet for them to do their exercise session—and where they can have their laptop/tablet webcam so as to be able to take part with best view for all
12.5—Adding objects to the environment	Thera-band and paper diaries and RPE scale
13.2—Framing/ Reframing	Suggest participants view intervention as a physical activity enabler rather than changing their pain
15.1—Verbal persuasion about capability	Positive reinforcement following initial assessment visit and in all sessions—intervention will be delivered in accordance to their abilities

## Discussion

The MRC framework for developing complex interventions brings focus on the need to understand and explicate what the active components are within an intervention, with theory development and context considered core elements [10]. The ToC development approach described here presents how the identification and review of the published evidence base and the understanding of the contextual issues around pain and exercise for PWH have been successfully integrated into synthesising a stakeholder informed programme theory for the development of a complex rehabilitation intervention. The resultant programme theory model is visually coherent and with the mapping of behaviour change theory to it, the stakeholders consider the theory to be plausible, credible and testable.

Complex interventions involve a number of interacting components that may require new behaviours by those receiving the intervention, or those who deliver it [22]. They may also have outcomes that are intended, unintended and multiple, and have implementation chains that can be long and convoluted [30]. It is this potential multitude and interlinking of known and unknown variables that creates the concept of the ‘black box’ in complex interventions, not just ‘what’ and ‘where’, but ‘why’ and ‘how’ observed effects may be taking place [31]. Living with a rare disorder such as severe haemophilia brings with it a complex medical regime needed to manage it, widespread musculoskeletal consequences of joint haemarthroses and the lived experience and beliefs of each individual. Acknowledging these multiple factors and the need to understand the degree of interplay between them, confirms that an exercise-based telerehabilitation intervention for PWH living with chronic pain can be considered a complex intervention.

Theory is a set of interrelated concepts and definitions that explain or predict events by specifying relationships between variables [32]. A programme theory describes how an intervention is expected to lead to its effects and under what conditions, and should articulate the key components and how they interact, and the relationship between the contextual influences and the mechanisms of interaction [10]. Rather than a more linear logic model, the outcomes chain diagram in a ToC places more focus on the causality through which the order of the activities is linked, thereby clearly identifying outcomes critical to success [33]. A well-articulated programme theory may optimise practice and provide accountability and efficiency for chosen interventions [34]. Relational detail such as that contained within the ToC model, can provide in-depth delivery knowledge and explanations informing whether the intervention needs to be modified for scaling up or used in different locations [35]. The ToC map created here identified that a trusting therapeutic relationship was key to accepting that this approach may be helpful in the overall delivery of the rehabilitation intervention. The stakeholders were very aware of the different social and physical contexts of potential participants with haemophilia and arthropathy, and as a result agreed the intervention should focus on low impact, whole body movement and activity, rather than be joint/limb specific. In highlighting this activity approach as an enabler, it was felt to be most likely to achieve the medium-term outcomes in confidence, knowledge, participation, and enjoyment of the programme.

Stakeholder engagement is a core element of the MRC framework in complex intervention development. Involvement of stakeholders from the outset is vital, as they understand the problem at hand and can identify the priorities in order to find realistic, workable and meaningful solutions [22]. Such an approach may be particularly worthwhile in rare conditions such as haemophilia where stakeholder involvement can improve interventions design and meaning as well as reducing research waste. Whilst there is little evidence in the current literature pertaining to involvement of PWH for intervention theory development, there have been other successful examples of participatory approaches in developing methods to improve haemophilia care delivery. Timmer and colleagues worked with stakeholders (PWH and primary care physiotherapists) to explore their experiences of primary care and develop recommendations to optimise physiotherapy care co-ordination [36]. In approaching this problem this way, they were able to get consensus on 13 recommendations for better physiotherapy care that may improve service quality and reduce waste. Similarly, a pain treatment planning questionnaire was conceptualised with PWH and carers. The tool was developed in partnership with patients interviewed to guide and inform the content, which was then further refined after clinical testing using a ‘Think aloud’ approach. The authors noted that the co-design approach was instrumental in developing the condition specific checklist within the questionnaire that was also acceptable for the patient population it was tested on [37]. Given the scale of potential benefits when inclusive stakeholder approaches are used, it is unfortunate there is little current evidence of such approaches being used to develop rehabilitation interventions for PWH.

The integration of behaviour change approaches in physiotherapy research and practice has been identified as a necessity to develop future interventions related to health promotion and wellbeing [38]. For any intervention that proposes to change behaviour, the UK National Institute for Health and Care Excellence (NICE) recommends that the content of the intervention is specified, detail is provided about what is done, by whom and in what context and it is clear what underlying theory will be used to make explicit the key causal links between actions and outcomes [39]. Whilst no studies in haemophilia to date have used this approach of mapping COM-B to qualitative findings, it has been identified as having potential importance in designing meaningful exercise based interventions for those with living with multi-morbidity [40]. A clearly documented development process is important to add to a growing body of knowledge about the explicit methods used in developing interventions [41], benefiting an understanding of the implementation as well as being able to examine the generalisability of the techniques used [24].

### Reflections on stakeholder engagement

The approach to stakeholder participation described here is novel in terms of previous approaches in PWH. With that in mind, it is important to include a reflective evaluation of their involvement in this process, and how their own interests and beliefs may have influenced and impacted the study.

All the PWH volunteers were white men, although they did have a wide age range and had a large diversity of experiences of haemophilia. One man had grown up in a country with minimal access to factor concentrate and comprehensive care, even when in adulthood. Another grew up with very intermittent and limited experiences of specialist physiotherapy. All of them had lived with pain associated with their haemophilia since childhood, but also had a positive view on the potential benefits of exercise. It was clear that all three men viewed their participation in this process as a philanthropic endeavour, viewing it as an opportunity to take part in something that might positively influence physiotherapy care provision for other PWH.

The two female physiotherapists who volunteered to be part of the stakeholder group were specialists from large treatment centres who had each worked in haemophilia for more than 10 years. Both had made contact with the lead author to volunteer their time for any projects associated with the study development. Similar to the PWH stakeholders, the physiotherapists did not expect a direct benefit from participating. They did note a desire to experience being part of an approach such as this, and an awareness that current approaches to pain management for PWH were insufficient.

The approach used in the theory of change workshop meant that all views were privileged as equal, enabling a safe and supported space for all suggestions to be talked through, and outcomes only reached by group discussion and group consensus. This focus kept the group coherent, and meant that the agreed suggestions had to be sensible and achievable in a real world setting, further adding to the impact of the theory of change model. The lead author moderated the workshop in the capacity as a critical friend, therefore sharing power and acknowledging all views to equally privileged.

This approach to stakeholder participation brought many benefits. It strengthened the focus on recruitment and delivery of the proposed telerehabilitation intervention, as well as highlighting which outcomes to evaluate within the study. The real world applicability of the proposed intervention, with a focus on a low impact/moderate intensity approach was probably the most impactful outcome of this process.

### Strengths and limitations

A major strength of this process is the transparency in the approach, with each step reflective of that which came before, and that which follows. The degree of detail regarding process means others can replicate it within their own environments, as well as being able to fully evaluate the process undertaken here.

The approach to co-production taken here for the ToC development is novel, but such an approach serves to shift the power dynamic away from the investigator and towards the stakeholders. The outcome of this has been a detailed, meaningful, and realistic theory that can be tested in real-world situations. The experiences and input of the stakeholders changed the intervention development for the better, and in doing so created a sense of ownership by them in that process.

Another strength is the clear, logical, informed process by which BCT’s were identified to be included in the study protocol. The BCT’s selection process can be situated in the synthesis and evaluation of the evidence base, as well as the mapping process onto the ToC co-produced by the stakeholders.

A limitation of this process may be the relatively small number of people involved in the ToC process. However, given the time and financial constraint associated with the development of a small feasibility study, the size of the stakeholder group was felt to be adequate by the research team. The ToC process itself was highly reflexive by virtue of the method and the review process of the map itself, thereby increasing transparency in the decisions made.

The stakeholders here in this study were adults currently receiving haemophilia care within the Healthcare system of the United Kingdom. Therefore, the output of the ToC may not be wholly transferable to paediatric populations, nor others in a different medical care system with differing geographical and contextual settings and experiences. However, the ToC method in itself is transferable for use in other haemophilia patient populations, and we encourage other researchers to consider its use when designing intervention studies.

Another limitation may be that the ToC map itself may be observed by others outside of the process to be lacking detail, or be thought to be missing outcome chains, activities, or enablers. This view is acceptable, but it must be remembered that the process described here is done in such way so as to be transparent and open to change. This iterative ability is what makes this approach advantageous for use in a feasibility study.

Whilst not a limitation, it should be highlighted that the lead author received specific training in the method, design and delivery of a Theory of Change workshop. Those interested in this method of stakeholder engagement should seek local providers of such training or expertise when thinking about this approach within their specific populations.

## Conclusion

The stakeholders in the theory of change process identified key outcomes within an interlinked causal model that were postulated to improve the credibility, testability, and acceptability of a proposed haemophilia telerehabilitation intervention. Behaviour change interventions were identified and mapped to the programme theory, with specific behaviour change techniques highlighted for inclusion in the intervention. The overall process involved a complex mix of evidence synthesis and evaluation against the requirement to inform a realistic and testable theory to underpin a study protocol. The result is a programme theory, described in detail, ready to be evaluated in a future feasibility study.

## Data Availability

Not applicable.

## Abbreviations

PWH: Person/people with haemophilia
ToC: Theory of change
MRC: Medical research council
BCT: Behaviour change technique
NICE: National Institute for Health and Care Excellence
